# Crystal structure of 4-{[(2,4-di­hydroxy­benzyl­idene)amino]­meth­yl}cyclo­hexane­carb­oxy­lic acid

**DOI:** 10.1107/S2056989015022343

**Published:** 2015-11-28

**Authors:** Muhammad Danish, Saba Akbar, Muhammad Nawaz Tahir, Rabia Ayub Butt, Muhammad Ashfaq

**Affiliations:** a Department of Chemistry, Institute of Natural Sciences, University of Gujrat, Gujrat 50700, Pakistan; b Department of physics, University of Sargodha, Sargodha, Punjab, Pakistan

**Keywords:** crystal structure, Schiff base, benzaldehyde, hydrogen bonding

## Abstract

In the title compound, C_15_H_19_NO_4_, the cyclo­hexyl ring adopts a chair conformation with both exocyclic C—C bonds in equatorial orientations. The dihedral angle between the basal plane of cyclo­hexyl ring and the 2,4-di­hydroxy­benzaldehyde moiety is 84.13 (13)°. An intra­molecular O—H⋯N hydrogen bonds closes an *S*(6) ring. In the crystal, O_c_—H⋯O_p_ (c = carb­oxy­lic acid, p = phenol) hydrogen bonds link the mol­ecules into [100] *C*(13) chains whereas an O_p_—H⋯O_c_ hydrogen bond generates [101] *C*(15) chains. Together, these bonds generate (010) sheets incorporating *R*_2_^2^(20) loops. Weak C—H⋯O and C—H⋯π inter­actions also occur.

## Related literature   

For the crystal structures of related Schiff bases, see: Shuja *et al.* (2006 ▸, 2007 ▸); Nisar *et al.* (2011 ▸).
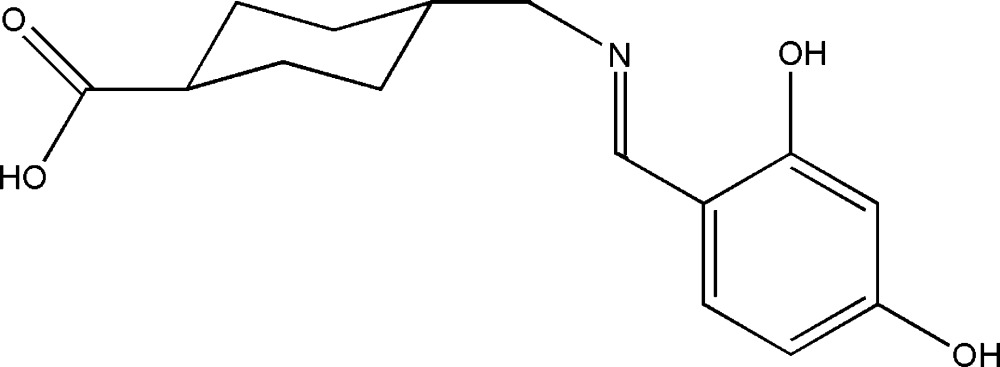


## Experimental   

### Crystal data  

C_15_H_19_NO_4_
*M* *_r_* = 277.31
Monoclinic, 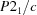

*a* = 6.2399 (17) Å
*b* = 10.222 (2) Å
*c* = 22.251 (6) Å
β = 90.232 (8)°
*V* = 1419.2 (6) Å^3^
*Z* = 4
Mo *K*α radiation
μ = 0.09 mm^−1^
*T* = 296 K
0.33 × 0.27 × 0.14 mm

### Data collection  

Bruker Kappa APEXII CCD diffractometer
Absorption correction: multi-scan (*SADABS*; Bruker, 2005 ▸) *T*_min_ = 0.970, *T*_max_ = 0.988
10670 measured reflections
2580 independent reflections
1222 reflections with *I* > 2σ(*I*)
*R*_int_ = 0.095

### Refinement  

*R*[*F* ^2^ > 2σ(*F* ^2^)] = 0.077
*wR*(*F* ^2^) = 0.214
*S* = 1.01
2580 reflections
186 parameters
H atoms treated by a mixture of independent and constrained refinement
Δρ_max_ = 0.35 e Å^−3^
Δρ_min_ = −0.26 e Å^−3^

### 

Data collection: *APEX2* (Bruker, 2007 ▸); cell refinement: *SAINT* (Bruker, 2007 ▸); data reduction: *SAINT*; program(s) used to solve structure: *SHELXS97* (Sheldrick, 2008 ▸); program(s) used to refine structure: *SHELXL2014*/6 (Sheldrick, 2015 ▸); molecular graphics: *ORTEP-3 for Windows* (Farrugia, 2012 ▸) and *PLATON* (Spek, 2009 ▸); software used to prepare material for publication: *WinGX* (Farrugia, 2012 ▸) and *PLATON*.

## Supplementary Material

Crystal structure: contains datablock(s) global, I. DOI: 10.1107/S2056989015022343/hb7548sup1.cif

Structure factors: contains datablock(s) I. DOI: 10.1107/S2056989015022343/hb7548Isup2.hkl

Supporting information file. DOI: 10.1107/S2056989015022343/hb7548Isup3.cml

**Fig. 1.**
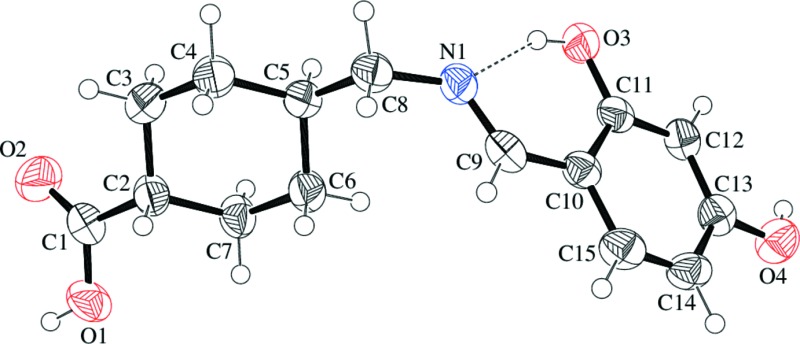
View of the title compound with displacement ellipsoids drawn at the 50% probability level. The dotted line represents the intramolecular hydrogen bonding.

. DOI: 10.1107/S2056989015022343/hb7548fig1.tif
View of the title compound with displacement ellipsoids drawn at the 50% probability level. The dotted line represents the intra­molecular hydrogen bonding.

**Fig. 2.**
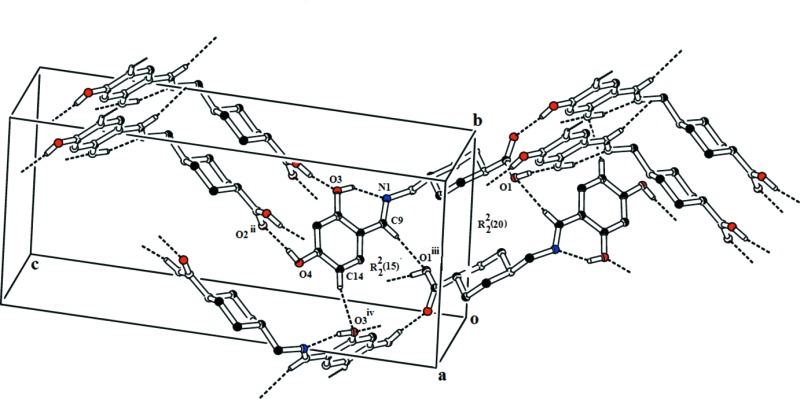
A partial packing diagram, showig that molecules form R22(20) and R33(15) ring motifs. H atoms not involved in hydrogen-bonding interactions are omitted for clarity.

. DOI: 10.1107/S2056989015022343/hb7548fig2.tif
A partial packing diagram, showig that mol­ecules form 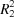
(20) and 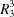
(15) ring motifs. H atoms not involved in hydrogen-bonding inter­actions are omitted for clarity.

CCDC reference: 1438286

Additional supporting information:  crystallographic information; 3D view; checkCIF report

## Figures and Tables

**Table 1:** Hydrogen-bond geometry (Å, °) *Cg*2 is the centroid of the C10–C15 benzene ring.

*D*—H⋯*A*	*D*—H	H⋯*A*	*D*⋯*A*	*D*—H⋯*A*
O1—H1⋯O3^i^	0.88 (5)	1.58 (5)	2.447 (4)	168 (5)
O3—H3⋯N1	0.82	1.92	2.667 (4)	150
O4—H4⋯O2^ii^	0.82	1.85	2.669 (4)	174
C9—H9⋯O1^iii^	0.93	2.42	3.338 (5)	170
C14—H14⋯O3^iv^	0.93	2.60	3.499 (5)	164
C5—H5⋯*Cg*2^v^	0.98	2.97	3.772 (5)	140

Symmetry codes: (i) 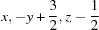; (ii) 
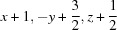
; (iii) 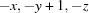; (iv) 
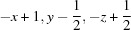
; (v) 
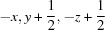
.

## supplementary crystallographic information

### S1. Comment

The title compound (I, Fig. 1) is the Schiff base ligand synthesized from tranexamic acid and 2,4-dihydroxybenzaldehyde. The reported crystal structures of the Schiff bases of tranexamic acid are 2-[(4-carboxycyclohexyl)methylammoniomethyl]-6-hydroxyphenolate (Shuja *et al.*, 2006), 4-((*E*)-(5-bromo-2-hydroxybenzylidene)aminomethyl)cyclohexane-1-carboxylic acid (Shuja *et al.*, 2007) and 4-({[(*E*)-pyridin-3-ylmethylidene]amino}-methyl)cyclohexanecarboxylic acid (Nisar *et al.*, 2011). The title compound is synthesized for various studies and complexation with different metals.

In (I), the basal plane A (C3/C4/C6/C7) of cyclohexyl ring and the part of 2,4-dihydroxybenzaldehyde B (C9—C14) are planar with r. m. s. deviation of 0.0101 Å and 0.0387 Å, respectively. The dihedral angle between A/B is 84.13 (13)°. The apical C-atoms C2 and C5 are almost at an equal distance of −0.680 (6) Å and 0.648 (6) Å, respectively from the plane A. The carboxylic part C (O1/C1/O2) is oriented at dihedral angles of 31.6 (3)° from planes A.

There exist *S*(6) ring motif due to O—H···N interactions (Table 1, Fig. 1). Each molecule is linked to four molecules due to O—H···O interactions (Table 1, Fig. 2) with *C*(13) and *C*(15) chains. *C*(13) chains exist from the 2-hydroxy and carboxyl hydroxy groups, where as *C*(15) chains are created when 4-hydroxy and carbonyl O-atom interlink. The C9—H9···O1^iii^ [iii = −*x*, −*y* + 1, −*z*] interactions generate *R*_2_^2^(20) ring motif (Table 1, Fig. 2). Similarly, the O4—H4···O2^ii^ [ii = *x* + 1, −*y* + 3/2, *z* + 1/2], C9—H9···O1^iii^ and C14—H14···O3^iv^ [iv = −*x* + 1, *y* − 1/2, −*z* + 1/2] interactions complete *R*_3_^3^(15) ring motifs. In this way, the alternate *R*_3_^3^(15) and *R*_2_^2^(20) ring motifs stabilize the molecules in the form of two-dimensional network with base vectors [1 0 0], [0 0 1] in the plane (0 1 0). A C—H···π interaction (Table 1) is also involved in the packing.

### S2. Experimental

Tranexamic acid (0.786 g, 5 mmol) and 2,4-dihydroxybenzaldehyde (0.661 g, 5 mmol) were disolved in 10 ml distilled water and 10 ml e thanol separately. These mixture were mixed and refluxed for 4 h to yield orange precipitate. The precipitates obtained were filtered and dried from which light orange plates of (I) were obtained after recrystallization in ethanol after one week.

Yield: 83%

Melting point:512 K.

### S3. Refinement

The coordinates of H-atoms of carboxylic acid were refined with constraints. The H-atoms were positioned geometrically (C–H = 0.93 − 0.98 Å, O–H = 0.82 Å) and refined as riding with *U*_iso_(H) = *xU*_eq_(C, O), where *x* = 1.5 for hydroxy and *x* = 1.2 for other H-atoms.

### Crystal data

**Table:**

C_15_H_19_NO_4_	*F*(000) = 592
*M**_r_* = 277.31	*D*_x_ = 1.298 Mg m^−^^3^
Monoclinic, *P*2_1_/*c*	Mo *K*α radiation, λ = 0.71073 Å
*a* = 6.2399 (17) Å	Cell parameters from 1222 reflections
*b* = 10.222 (2) Å	θ = 2.7–25.3°
*c* = 22.251 (6) Å	µ = 0.09 mm^−^^1^
β = 90.232 (8)°	*T* = 296 K
*V* = 1419.2 (6) Å^3^	Plate, light orange
*Z* = 4	0.33 × 0.27 × 0.14 mm

### Data collection

**Table:**

Bruker Kappa APEXII CCD diffractometer	2580 independent reflections
Radiation source: fine-focus sealed tube	1222 reflections with *I* > 2σ(*I*)
Graphite monochromator	*R*_int_ = 0.095
Detector resolution: 7.80 pixels mm^-1^	θ_max_ = 25.3°, θ_min_ = 2.7°
ω scans	*h* = −7→7
Absorption correction: multi-scan (*SADABS*; Bruker, 2005)	*k* = −12→8
*T*_min_ = 0.970, *T*_max_ = 0.988	*l* = −27→27
10670 measured reflections	

### Refinement

**Table:**

Refinement on *F*^2^	Primary atom site location: structure-invariant direct methods
Least-squares matrix: full	Secondary atom site location: difference Fourier map
*R*[*F*^2^ > 2σ(*F*^2^)] = 0.077	Hydrogen site location: inferred from neighbouring sites
*wR*(*F*^2^) = 0.214	H atoms treated by a mixture of independent and constrained refinement
*S* = 1.01	*w* = 1/[σ^2^(*F*_o_^2^) + (0.0891*P*)^2^] where *P* = (*F*_o_^2^ + 2*F*_c_^2^)/3
2580 reflections	(Δ/σ)_max_ < 0.001
186 parameters	Δρ_max_ = 0.35 e Å^−^^3^
0 restraints	Δρ_min_ = −0.26 e Å^−^^3^

### Special details

**Table:**

Geometry. All e.s.d.'s (except the e.s.d. in the dihedral angle between two l.s. planes) are estimated using the full covariance matrix. The cell e.s.d.'s are taken into account individually in the estimation of e.s.d.'s in distances, angles and torsion angles; correlations between e.s.d.'s in cell parameters are only used when they are defined by crystal symmetry. An approximate (isotropic) treatment of cell e.s.d.'s is used for estimating e.s.d.'s involving l.s. planes.
Refinement. Refinement of **F**^2^ against ALL reflections. The weighted **R**-factor *wR* and goodness of fit **S** are based on **F**^2^, conventional **R**-factors **R** are based on **F**, with **F** set to zero for negative **F**^2^. The threshold expression of **F**^2^ > σ(**F**^2^) is used only for calculating **R**-factors(gt) *etc*. and is not relevant to the choice of reflections for refinement. **R**-factors based on **F**^2^ are statistically about twice as large as those based on **F**, and **R**-factors based on ALL data will be even larger.

### Fractional atomic coordinates and isotropic or equivalent isotropic displacement parameters (Å^2^)

**Table:**

	*x*	*y*	*z*	*U*_iso_*/*U*_eq_	
O1	0.0521 (6)	0.7966 (3)	−0.09932 (14)	0.0576 (10)	
H1	0.082 (8)	0.840 (5)	−0.132 (2)	0.086*	
O2	−0.2047 (6)	0.9366 (3)	−0.07558 (15)	0.0717 (11)	
O3	0.0919 (5)	0.5993 (3)	0.30231 (12)	0.0465 (8)	
H3	0.0041	0.6040	0.2749	0.070*	
O4	0.7509 (5)	0.3799 (3)	0.33996 (14)	0.0549 (9)	
H4	0.7673	0.4399	0.3640	0.082*	
N1	−0.1192 (6)	0.5339 (3)	0.20228 (15)	0.0426 (10)	
C1	−0.1074 (8)	0.8366 (4)	−0.06681 (19)	0.0441 (11)	
C2	−0.1625 (7)	0.7441 (4)	−0.01567 (18)	0.0430 (11)	
H2	−0.1976	0.6593	−0.0337	0.052*	
C3	−0.3547 (7)	0.7867 (5)	0.0196 (2)	0.0604 (14)	
H3A	−0.4763	0.7979	−0.0072	0.072*	
H3B	−0.3256	0.8700	0.0389	0.072*	
C4	−0.4078 (7)	0.6832 (5)	0.06750 (19)	0.0563 (13)	
H4A	−0.5306	0.7123	0.0905	0.068*	
H4B	−0.4464	0.6020	0.0477	0.068*	
C5	−0.2214 (6)	0.6584 (4)	0.10995 (18)	0.0425 (11)	
H5	−0.1913	0.7394	0.1320	0.051*	
C6	−0.0247 (7)	0.6216 (4)	0.07511 (18)	0.0498 (12)	
H6A	−0.0470	0.5367	0.0566	0.060*	
H6B	0.0958	0.6142	0.1026	0.060*	
C7	0.0299 (7)	0.7226 (4)	0.02570 (18)	0.0470 (12)	
H7A	0.0706	0.8049	0.0442	0.056*	
H7B	0.1505	0.6913	0.0024	0.056*	
C8	−0.2828 (7)	0.5528 (4)	0.15533 (18)	0.0454 (12)	
H8A	−0.4172	0.5768	0.1741	0.054*	
H8B	−0.3048	0.4708	0.1343	0.054*	
C9	0.0253 (7)	0.4422 (4)	0.19944 (18)	0.0418 (11)	
H9	0.0123	0.3825	0.1681	0.050*	
C10	0.1980 (7)	0.4246 (4)	0.23882 (18)	0.0377 (11)	
C11	0.2338 (7)	0.5082 (3)	0.28987 (19)	0.0388 (11)	
C12	0.4181 (7)	0.4917 (4)	0.32423 (18)	0.0407 (11)	
H12	0.4411	0.5447	0.3576	0.049*	
C13	0.5676 (8)	0.3976 (4)	0.30947 (19)	0.0419 (11)	
C14	0.5317 (8)	0.3123 (4)	0.26061 (19)	0.0460 (12)	
H14	0.6298	0.2467	0.2516	0.055*	
C15	0.3522 (7)	0.3274 (4)	0.2270 (2)	0.0466 (12)	
H15	0.3296	0.2712	0.1947	0.056*	

### Atomic displacement parameters (Å^2^)

**Table:**

	*U*^11^	*U*^22^	*U*^33^	*U*^12^	*U*^13^	*U*^23^
O1	0.077 (3)	0.055 (2)	0.041 (2)	0.0053 (17)	0.0165 (19)	0.0106 (15)
O2	0.078 (3)	0.070 (2)	0.068 (2)	0.026 (2)	0.014 (2)	0.0257 (19)
O3	0.054 (2)	0.0480 (18)	0.0371 (18)	0.0076 (16)	−0.0024 (15)	−0.0005 (14)
O4	0.052 (2)	0.053 (2)	0.059 (2)	0.0024 (16)	−0.0121 (18)	−0.0066 (16)
N1	0.042 (2)	0.049 (2)	0.037 (2)	−0.0004 (18)	0.0041 (19)	0.0091 (17)
C1	0.051 (3)	0.045 (3)	0.036 (3)	−0.005 (2)	−0.002 (2)	0.001 (2)
C2	0.044 (3)	0.050 (3)	0.036 (2)	−0.001 (2)	−0.003 (2)	0.006 (2)
C3	0.049 (3)	0.081 (3)	0.051 (3)	0.021 (3)	0.004 (3)	0.011 (3)
C4	0.031 (3)	0.091 (4)	0.046 (3)	0.004 (3)	0.006 (2)	0.013 (3)
C5	0.039 (3)	0.051 (3)	0.038 (3)	0.004 (2)	0.001 (2)	0.000 (2)
C6	0.045 (3)	0.064 (3)	0.040 (3)	0.005 (2)	0.002 (2)	0.012 (2)
C7	0.038 (3)	0.063 (3)	0.040 (3)	−0.004 (2)	−0.002 (2)	0.009 (2)
C8	0.041 (3)	0.054 (3)	0.042 (3)	0.000 (2)	0.005 (2)	0.005 (2)
C9	0.049 (3)	0.044 (3)	0.032 (3)	−0.010 (2)	0.003 (2)	−0.001 (2)
C10	0.044 (3)	0.035 (2)	0.035 (2)	−0.003 (2)	0.003 (2)	−0.0015 (19)
C11	0.053 (3)	0.027 (2)	0.037 (3)	−0.001 (2)	0.012 (2)	0.0063 (19)
C12	0.053 (3)	0.034 (2)	0.035 (3)	−0.005 (2)	−0.005 (2)	−0.0015 (19)
C13	0.049 (3)	0.034 (2)	0.043 (3)	−0.006 (2)	0.000 (2)	0.003 (2)
C14	0.054 (3)	0.032 (2)	0.052 (3)	0.002 (2)	0.010 (3)	−0.006 (2)
C15	0.056 (3)	0.036 (2)	0.047 (3)	−0.005 (2)	0.003 (3)	−0.010 (2)

### Geometric parameters (Å, º)

**Table:**

O1—C1	1.299 (5)	C5—C8	1.528 (5)
O1—H1	0.88 (5)	C5—H5	0.9800
O2—C1	1.205 (5)	C6—C7	1.547 (5)
O3—C11	1.316 (4)	C6—H6A	0.9700
O3—H3	0.8200	C6—H6B	0.9700
O4—C13	1.340 (5)	C7—H7A	0.9700
O4—H4	0.8200	C7—H7B	0.9700
N1—C9	1.303 (5)	C8—H8A	0.9700
N1—C8	1.470 (5)	C8—H8B	0.9700
C1—C2	1.520 (5)	C9—C10	1.398 (6)
C2—C3	1.501 (5)	C9—H9	0.9300
C2—C7	1.526 (6)	C10—C15	1.409 (5)
C2—H2	0.9800	C10—C11	1.438 (5)
C3—C4	1.538 (6)	C11—C12	1.388 (6)
C3—H3A	0.9700	C12—C13	1.381 (5)
C3—H3B	0.9700	C12—H12	0.9300
C4—C5	1.517 (6)	C13—C14	1.411 (5)
C4—H4A	0.9700	C14—C15	1.353 (6)
C4—H4B	0.9700	C14—H14	0.9300
C5—C6	1.502 (5)	C15—H15	0.9300
			
C1—O1—H1	118 (3)	C7—C6—H6B	109.1
C11—O3—H3	109.5	H6A—C6—H6B	107.8
C13—O4—H4	109.5	C2—C7—C6	110.5 (3)
C9—N1—C8	122.6 (4)	C2—C7—H7A	109.6
O2—C1—O1	124.3 (4)	C6—C7—H7A	109.6
O2—C1—C2	122.3 (4)	C2—C7—H7B	109.6
O1—C1—C2	113.4 (4)	C6—C7—H7B	109.6
C3—C2—C1	113.2 (4)	H7A—C7—H7B	108.1
C3—C2—C7	110.8 (4)	N1—C8—C5	112.8 (3)
C1—C2—C7	111.2 (3)	N1—C8—H8A	109.0
C3—C2—H2	107.1	C5—C8—H8A	109.0
C1—C2—H2	107.1	N1—C8—H8B	109.0
C7—C2—H2	107.1	C5—C8—H8B	109.0
C2—C3—C4	109.7 (4)	H8A—C8—H8B	107.8
C2—C3—H3A	109.7	N1—C9—C10	126.5 (4)
C4—C3—H3A	109.7	N1—C9—H9	116.8
C2—C3—H3B	109.7	C10—C9—H9	116.8
C4—C3—H3B	109.7	C9—C10—C15	119.9 (4)
H3A—C3—H3B	108.2	C9—C10—C11	122.4 (4)
C5—C4—C3	112.3 (4)	C15—C10—C11	117.5 (4)
C5—C4—H4A	109.1	O3—C11—C12	121.8 (4)
C3—C4—H4A	109.1	O3—C11—C10	119.0 (4)
C5—C4—H4B	109.1	C12—C11—C10	119.3 (4)
C3—C4—H4B	109.1	C13—C12—C11	120.8 (4)
H4A—C4—H4B	107.9	C13—C12—H12	119.6
C6—C5—C4	110.3 (3)	C11—C12—H12	119.6
C6—C5—C8	111.8 (3)	O4—C13—C12	123.4 (4)
C4—C5—C8	109.6 (3)	O4—C13—C14	116.1 (4)
C6—C5—H5	108.3	C12—C13—C14	120.6 (4)
C4—C5—H5	108.3	C15—C14—C13	118.9 (4)
C8—C5—H5	108.3	C15—C14—H14	120.5
C5—C6—C7	112.5 (3)	C13—C14—H14	120.5
C5—C6—H6A	109.1	C14—C15—C10	122.8 (4)
C7—C6—H6A	109.1	C14—C15—H15	118.6
C5—C6—H6B	109.1	C10—C15—H15	118.6
			
O2—C1—C2—C3	3.2 (6)	C8—N1—C9—C10	173.6 (4)
O1—C1—C2—C3	−176.7 (4)	N1—C9—C10—C15	−174.5 (4)
O2—C1—C2—C7	−122.3 (5)	N1—C9—C10—C11	1.4 (6)
O1—C1—C2—C7	57.8 (5)	C9—C10—C11—O3	4.4 (6)
C1—C2—C3—C4	176.2 (4)	C15—C10—C11—O3	−179.5 (3)
C7—C2—C3—C4	−58.1 (5)	C9—C10—C11—C12	−174.8 (4)
C2—C3—C4—C5	57.8 (5)	C15—C10—C11—C12	1.3 (5)
C3—C4—C5—C6	−55.2 (5)	O3—C11—C12—C13	−178.3 (3)
C3—C4—C5—C8	−178.7 (3)	C10—C11—C12—C13	0.8 (6)
C4—C5—C6—C7	53.5 (5)	C11—C12—C13—O4	178.0 (4)
C8—C5—C6—C7	175.7 (4)	C11—C12—C13—C14	−2.8 (6)
C3—C2—C7—C6	56.7 (5)	O4—C13—C14—C15	−178.2 (4)
C1—C2—C7—C6	−176.5 (4)	C12—C13—C14—C15	2.5 (6)
C5—C6—C7—C2	−54.6 (5)	C13—C14—C15—C10	−0.3 (6)
C9—N1—C8—C5	−96.8 (5)	C9—C10—C15—C14	174.6 (4)
C6—C5—C8—N1	63.9 (5)	C11—C10—C15—C14	−1.5 (6)
C4—C5—C8—N1	−173.5 (3)		

### Hydrogen-bond geometry (Å, º)

Cg2 is the centroid of the C10–C15 benzene ring.

**Table:**

*D*—H···*A*	*D*—H	H···*A*	*D*···*A*	*D*—H···*A*
O1—H1···O3^i^	0.88 (5)	1.58 (5)	2.447 (4)	168 (5)
O3—H3···N1	0.82	1.92	2.667 (4)	150
O4—H4···O2^ii^	0.82	1.85	2.669 (4)	174
C9—H9···O1^iii^	0.93	2.42	3.338 (5)	170
C14—H14···O3^iv^	0.93	2.60	3.499 (5)	164
C5—H5···*Cg*2^v^	0.98	2.97	3.772 (5)	140

Symmetry codes: (i) *x*, −*y*+3/2, *z*−1/2; (ii) *x*+1, −*y*+3/2, *z*+1/2; (iii) −*x*, −*y*+1, −*z*; (iv) −*x*+1, *y*−1/2, −*z*+1/2; (v) −*x*, *y*+1/2, −*z*+1/2.
