# Supplementary figures and images for: Crystal structure of 4-{[(2,4-di­hydroxy­benzyl­idene)amino]­meth­yl}cyclo­hexane­carb­oxy­lic acid
Source: Acta Crystallogr E Crystallogr Commun. 2015 Nov 28;71(Pt 12):o995–6. doi: 10.1107/S2056989015022343 (PMC4719941; doi:10.1107/S2056989015022343)

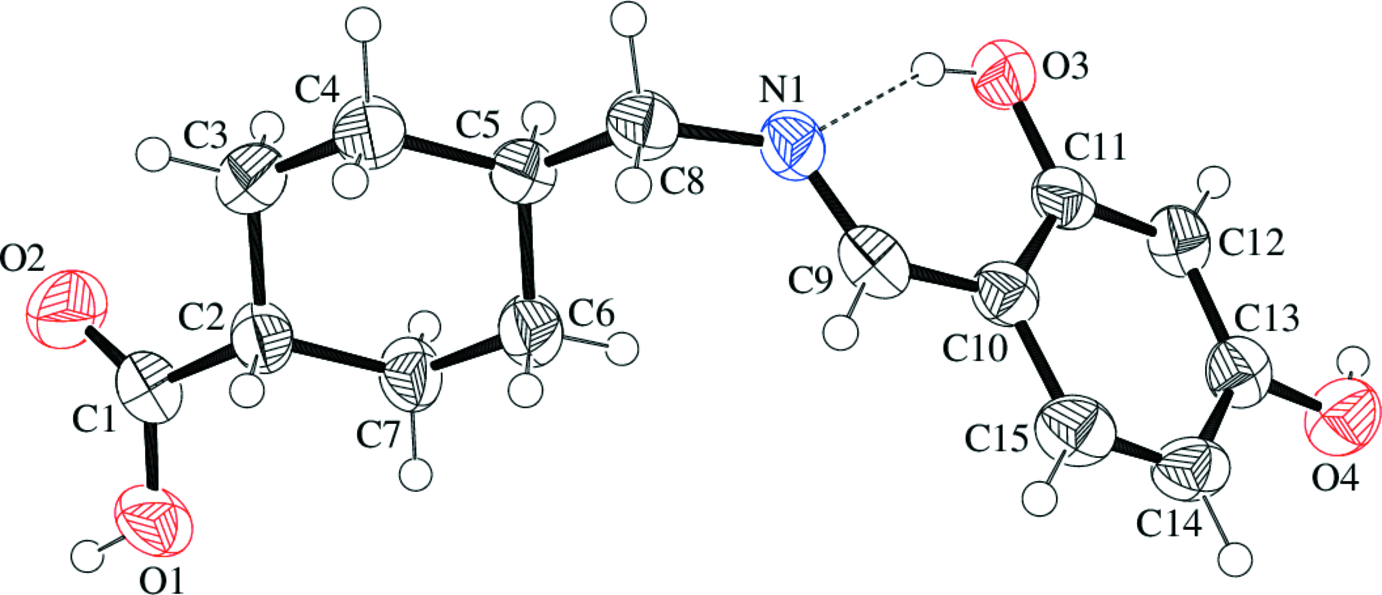

Supplement: Supplementary file 4 [file e-71-0o995-fig1.tif]

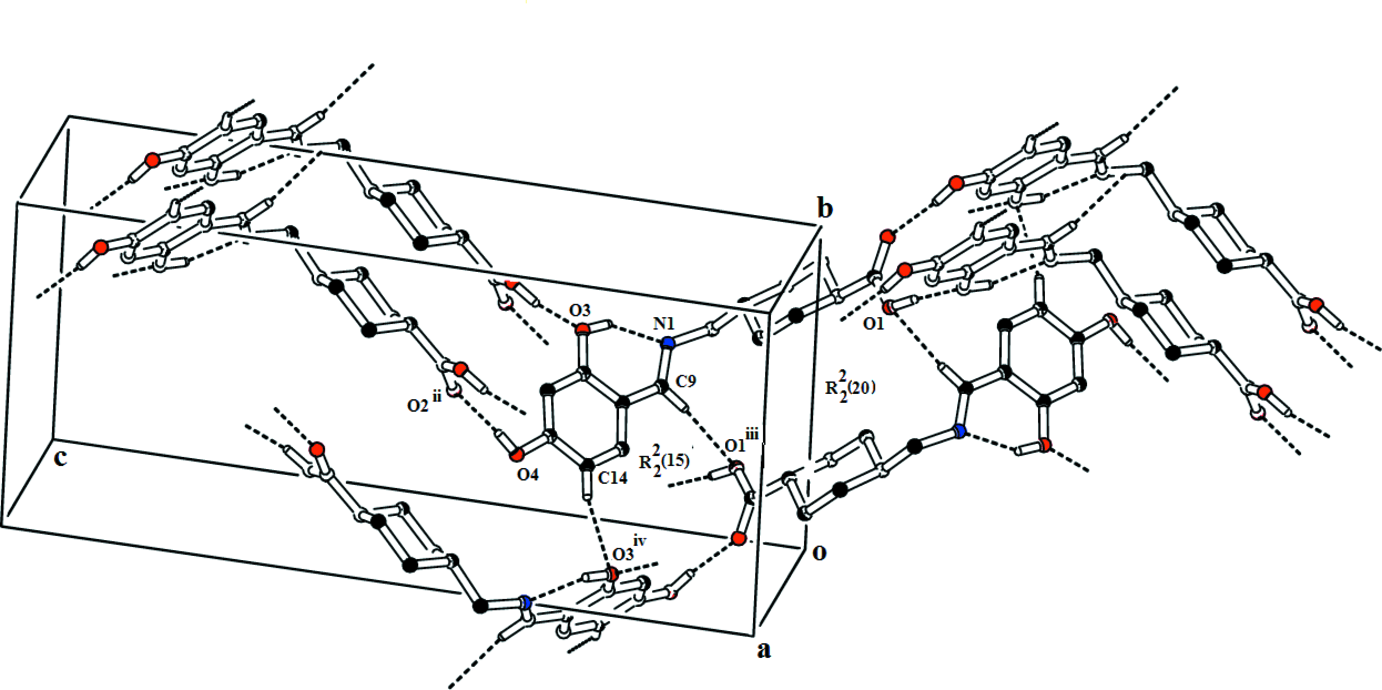

Supplement: Supplementary file 5 [file e-71-0o995-fig2.tif]
